# Sustainable Protein Transitions: A Comprehensive Review of Insect Meal in Broiler Diets—Nutritional Value, Immunomodulatory Effects, Gut Health Interactions, and Future Perspectives

**DOI:** 10.3390/biology15151320

**Published:** 2026-08-06

**Authors:** Osama K. Abou-Emera

**Affiliations:** 1 Department of Animal and Poultry Production, College of Agriculture and Food, Qassim University, Buraydah 51452, Saudi Arabia; o.emara@qu.edu.sa; 2 Department of Poultry Breeding Research, Animal Production Research Institute, Agriculture Research Centre, Dokki, Giza 12618, Egypt

**Keywords:** black soldier fly larvae, *Hermetia illucens*, broiler nutrition, immunomodulation, gut microbiome, sustainable protein, antimicrobial peptides, chitin

## Abstract

Broiler production keeps intensifying, and the environmental cost of the protein sources that feed it, principally soybean meal and fishmeal, has become harder to justify. Insect meal, derived chiefly from black soldier fly larvae (*Hermetia illucens*), yellow mealworm (*Tenebrio molitor*), and housefly larvae (*Musca domestica*), has emerged as a candidate able to address nutritional, immunological, and ecological goals at once. This review evaluates the evidence on the nutritional composition, digestibility, growth performance, immunomodulatory mechanisms, and gut health effects of insect meal in broiler diets, and weighs these findings against sustainability and economic considerations. The amino acid profile of insect meal is favorable, the fatty acid composition is distinctive, and bioactive compounds, chitin, antimicrobial peptides, and lauric acid among them, shape both innate and adaptive immune responses, reshape the gut microbiota, and reinforce mucosal integrity. Growth performance at partial replacement levels of soybean meal or fishmeal (5–15%) is broadly encouraging, although results differ across insect species, inclusion rate, and processing method. The immunomodulatory pathways involved, Toll-like receptor signaling, cytokine regulation, macrophage polarization, and immunoglobulin synthesis, converge on chitin acting simultaneously as a prebiotic substrate and an immune adjuvant. Critical knowledge gaps persist, including substantial variability in biological outcomes across insect species and rearing substrates, the absence of standardized processing protocols, limited mechanistic data from avian in vivo challenge models, and insufficient long-term validation under commercial production conditions. Cost and regulatory fragmentation across jurisdictions still constrain commercial uptake. Realizing the full potential of insect meal in sustainable broiler production will depend on multi-omics research, precision nutrition tools, and rigorous life-cycle assessment.

## Simple Summary

Broiler diets primarily rely on soybean meal and fishmeal, both of which carry significant environmental costs. Insect meal, principally from black soldier fly larvae (*Hermetia illucens*), is one of the few alternatives that can reasonably close that gap. Beyond supplying a strong amino acid profile, insect-derived ingredients bring lauric acid and chitin to the table, compounds that plant and marine proteins simply do not have, and both appear to support gut health and immune function in ways that go beyond ordinary protein replacement. Feeding trials replacing 5–15% of dietary soybean or fishmeal with insect meal consistently maintain body weight gain and feed conversion ratio within approximately 5% of conventional diet controls; at 10% BSF larval meal inclusion, several trials report numerically superior performance relative to SBM-based diets. At the environmental level, insect production systems can reduce greenhouse gas emissions by more than 80% relative to fishmeal under circular economy conditions. What remains unresolved is consensus on processing standards, dosing, and the applicability of laboratory findings to commercial settings. Insect meal currently remains more expensive than soybean meal, but cost trajectories point toward narrowing competitiveness at industrial scale. In summary, the evidence supports insect meal as a scientifically credible and ecologically coherent component of a more sustainable broiler sector.

## 1. Introduction

Global poultry production has expanded at an extraordinary pace over the past four decades, driven by population growth, urbanization, rising middle-class incomes across Asia, Africa, and Latin America, and the comparatively light environmental footprint of poultry relative to ruminant livestock. Broiler chicken is now the single most consumed animal protein worldwide: annual production already exceeds 130 million metric tons and is projected to climb a further 25–30% by 2050 [1]. According to FAO projections, poultry meat demand in developing regions alone is expected to increase by more than 30% by 2030, making the security of sustainable, scalable protein inputs an urgent priority across global food systems [1,2].

Soybean meal (SBM) and fishmeal (FM) have long anchored commercial broiler diets, supplying essential amino acids in ratios that suit avian requirements reasonably well [2,3]. Both, however, come with ecological costs that are increasingly hard to square with sustainability goals. Soybean cultivation, concentrated in South America and dominated by Brazilian and Argentinian exports, has repeatedly been linked to deforestation in the Amazon and Cerrado, greenhouse gas emissions, freshwater depletion, and biodiversity loss [4,5]. Estimates put the carbon footprint of soybean meal at roughly 2.0–3.0 kg CO_2_-eq per kilogram of protein, a figure that climbs considerably once land-use change is factored in. Fishmeal, drawn mainly from anchovy, menhaden, and sardine fisheries, adds pressure to already stressed pelagic fish stocks and carries a carbon footprint of 3.0–12.0 kg CO_2_-eq per kilogram, depending on where and how it is caught [6]. Alongside SBM and FM, other alternative protein sources under active investigation include processed animal proteins, microalgae, and single-cell proteins. However, these alternatives each carry significant constraints: processed animal proteins face stringent regulatory limitations in many markets; microalgae production remains expensive and energy-intensive at scale; and single-cell proteins require specialized fermentation infrastructure. Insect meal’s capacity to simultaneously valorize organic waste streams, produce high-quality protein together with functional bioactive compounds, and integrate into existing agricultural supply chains constitutes a distinct set of advantages over all of these alternatives.

The idea of a circular bioeconomy—in which nutrient flows loop back into the system, waste streams gain value, and ecological externalities shrink—has gained real traction among policymakers, industry, and academia as a way of redesigning food and feed systems [7]. Insect farming fits neatly into that picture. Insects can be reared on organic side-streams, agricultural co-products, food waste, and processing residues, turning materials with little nutritional value for humans into high-quality protein and lipid biomass, and doing so with a fraction of the greenhouse gas output, land, and water that conventional livestock protein demands [8]. When the European Commission revised its feed legislation in 2021 to permit processed animal proteins from insects in poultry and pig feed, it removed a major regulatory bottleneck and triggered a wave of industrial investment and research activity across Europe and beyond.

Research on insect meal as a broiler feed ingredient has grown quickly over the past decade. The earliest studies were largely descriptive: establishing baseline nutrient profiles and confirming that growth performance held up under partial replacement. More recent work has pushed further, probing the mechanisms behind the responses observed, particularly the immunomodulatory, prebiotic, and antimicrobial properties of insect-derived bioactives, with implications reaching well past simple protein substitution. Chitin, antimicrobial peptides (AMPs), lauric acid, and other functional lipids give insect meal a biochemical complexity that plant and marine protein sources simply do not have, which is why it makes more sense to treat it as a functional feed ingredient in its own right than as just another protein carrier.

This review was conducted following a systematic literature search of the PubMed, Scopus, and Web of Science databases using the search terms insect meal, *Hermetia illucens*, *Tenebrio molitor*, and *Musca domestica* individually and in combination. The search covered publications from 2000 to 2026, with priority given to peer-reviewed original research articles and review papers published from 2018 onwards. Seminal studies from before 2018 were retained where they represent foundational contributions unavailable in more recent form.

Nevertheless, the literature is far from tidy. Findings on inclusion thresholds, digestibility coefficients, immune biomarker responses, and microbiota shifts often conflict, and much of that disagreement reflects genuine biological complexity: insect species, rearing substrate, larval stage, processing method (defatted versus full-fat, dried versus hydrolyzed), bird genotype, age, and basal diet composition all shift outcomes enough that comparing studies at face value is risky. Small sample sizes, short trial durations, and an almost total absence of multi-site validation compound the problem.

What distinguishes this review from the growing body of insect meal literature is its integrative approach: rather than treating nutrition, immunity, gut microbiota, and sustainability as parallel domains, it connects these dimensions into a single mechanistic framework and maps their interactions systematically. Recent narrative reviews have begun to synthesize specific aspects of insect meal biology; for example, Naeem [9] integrates nutritional value, functional bioactive compounds, gut health, immune modulation, sustainability, and commercial challenges in a complementary overview. The present manuscript builds on this landscape by providing detailed mechanistic analysis of immunomodulatory signaling pathways, critical evaluation of conflicting evidence across studies, and a structured research agenda for the next decade, with the explicit aim of connecting insect production systems, bioactive compound profiles, gut health dynamics, immune modulation, growth performance, and sustainability outcomes into one coherent framework.

## 2. Insect Production Systems and Sustainability

### 2.1. Overview of Major Insect Species in Feed Production

Several insect species have drawn serious research and commercial interest, but three dominate current practice: *Hermetia illucens* (black soldier fly, BSF), *Tenebrio molitor* (yellow mealworm, YMW), and *Musca domestica* (housefly). A smaller group, including *Acheta domesticus* (house cricket), *Gryllus bimaculatus* (field cricket), *Locusta migratoria* (migratory locust), and *Bombyx mori* (silkworm), has found regional footholds without reaching comparable commercial scale. Each species brings its own combination of biological traits, substrate tolerance, nutritional output, and scalability, and that combination decides how well it fits the circular bioeconomy model.

*Hermetia illucens* has become the leading industrial species, mostly because it thrives on low-quality organic substrates, food waste, animal manure, agricultural residues, brewing by-products, and develops from egg to harvestable larva in just 14–22 days under good conditions [10]. BSF larvae do not need moisture-rich substrate to pupate, tolerate heavy microbial loads, and yield crude protein concentrations of 35–45% with fat contents of 25–40% on a dry matter basis, the exact numbers depending on substrate and harvest timing [3,11]. Because adult flies do not feed, BSF rearing does not compete with human food systems in the way some other protein sources do; the larvae simply consume waste that would otherwise need costly disposal or processing. Life-cycle assessments consistently find that BSF meal generates far fewer greenhouse gas emissions per unit of protein than soybean or fishmeal, though the exact figures shift with rearing system design, location, and energy source [12,13].

An important safety limitation for commercial adoption concerns the potential accumulation of heavy metals, pesticides, and veterinary drug residues in BSF larvae, which depends critically on the composition of the rearing substrate. When larvae are cultivated on substrates contaminated with heavy metals (certain municipal food waste streams, compost materials, or agricultural co-products processed with pesticides) accumulation of cadmium, lead, and copper in larval biomass has been documented, with concentrations in some cases approaching or exceeding EU maximum limits for feed ingredients. Similarly, veterinary drug residues and persistent organic pesticides present in manure-based substrates can transfer into larval tissue. The 2021 EU regulation (EU 2017/893, as amended) directly addresses this risk by restricting commercially permitted substrates to materials of verified safe provenance; however, the risk remains practically relevant in production systems operating outside regulated frameworks, and substrate quality monitoring remains an under-standardized area requiring greater regulatory and scientific attention.

*Tenebrio molitor* occupies different ground. Mealworm larvae are pickier about substrate, favoring cereal brans, grain by-products, and the organic fraction of municipal waste, inputs that, unlike much of what feeds BSF, compete to some degree with human food uses. What YMW gives up in substrate flexibility it partly makes up for in amino acid quality, with methionine and cysteine levels notably higher than in BSF [14]. Production has historically lagged behind BSF because of processing and scaling constraints, though automated rearing and tighter environmental control have narrowed that gap considerably. A favorable safety assessment from the European Food Safety Authority for *T. molitor* as a novel food and feed ingredient [15] has since sped up regulatory approvals across EU member states.

*Musca domestica* has been studied most extensively in Asian production systems, particularly in China, where the research infrastructure and regulatory landscape differ markedly from Europe’s. Housefly larvae can be reared on swine and poultry manure, which allows tight integration with existing livestock operations, but that same substrate flexibility raises biosecurity concerns: manure-reared insects may carry zoonotic pathogens or accumulate antibiotic residues, complicating safety assessment and regulatory approval in stricter markets. Even so, housefly larval meal (HLM) has shown nutritional and immunostimulatory promise across multiple broiler trials, making it scientifically informative even where commercial rollout faces headwinds.

### 2.2. Resource Efficiency and Environmental Performance

Insect farming’s clearest environmental advantage is feed conversion efficiency. A kilogram of beef requires roughly 8–10 kg of feed on a live-weight basis, and pigs need 4–5 kg, while insects convert organic substrate into biomass at ratios of just 1.5–2.5 kg substrate per kilogram of larval fresh weight [2]. Land use tells a similar story: producing insect protein takes an estimated 10 to 70 times less land per unit of protein than soybean cultivation or conventional livestock [13]. Water footprints follow suit. BSF production uses about 1–4 L of water per kilogram of protein, against 112 L for soybean meal and up to 5990 L for beef protein [12,16].

Life-cycle studies of greenhouse gas emissions tell the same story from a different angle. Oonincx and de Boer [12] found that *T. molitor* production generated 2850 g CO_2_-eq per kilogram of body mass gain, well below pork or beef and roughly on par with, or better than, chicken by some measures. Rearing insects on organic waste improves the accounting further, since waste diversion earns a carbon credit of its own. That said, LCA methodologies vary enough in system boundaries, allocation rules, and impact categories that comparing studies directly can be misleading. Energy use in rearing facilities, especially climate control, lighting, and substrate processing, tends to be the dominant environmental hotspot across most insect production systems, and it is where technological improvement would pay off most [17].

### 2.3. Circular Bioeconomy Integration and Waste Valorization

What makes insect farming genuinely compelling at a systems level is how naturally it fits circular food and feed models. The circular bioeconomy concept holds that biological resources should stay in circulation at their highest possible value, with linear flows and outside inputs kept to a minimum [7]. Insect rearing on organic waste checks several of those boxes simultaneously: it converts nutrient-rich side-streams that would otherwise need disposal, or incomplete recycling, into high-value protein and lipid biomass, while cutting the volume and pathogen load of the leftover frass, a by-product already valued as a soil amendment and fertilizer [18]. That frass carries meaningful nitrogen, phosphorus, and chitin content, pointing to real potential in both horticulture and agriculture. Increasingly, the economic and ecological case for insect farming rests on using the whole biorefinery output, protein, lipid, chitin, and frass, rather than treating dried larval meal as the only product worth counting.

**Figure 1:**
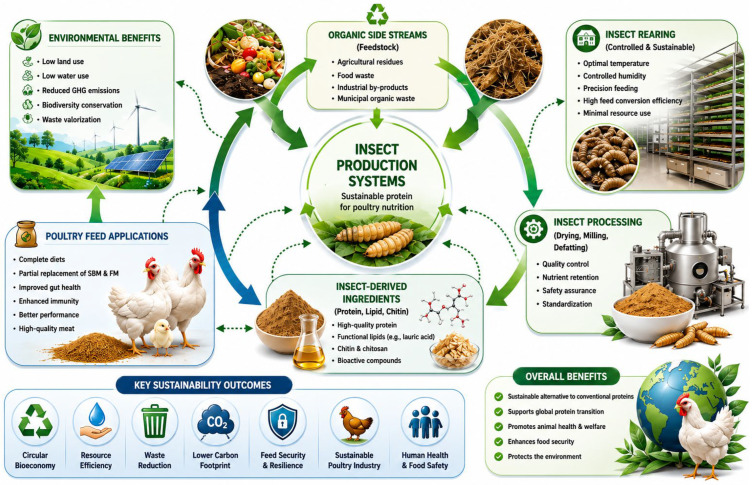
Insect Production Systems and Sustainability: a circular bioeconomy approach for sustainable poultry nutrition.

Set against fishmeal and soybean meal, insect meal’s sustainability profile looks favorable on the whole, though it is emphatically not unconditional. The life-cycle environmental advantage of insect production depends heavily on two factors: the origin and quality of the rearing substrate, and the energy source powering the rearing facility. Powered by renewable energy and built on genuine organic waste streams, insect production can cut greenhouse gas emissions relative to fishmeal by more than 80%. Rear the same insects on dedicated grain crops in fossil-fuel-powered facilities with energy-intensive climate control, however, and that advantage shrinks considerably or may even disappear on some environmental metrics [13,17]. These boundary conditions underscore that context-specific life-cycle analysis matters, and that blanket claims of insect meal’s universal sustainability cannot be made without transparent reporting of substrate origin, energy mix, and system boundaries. A summary of insect production systems and their sustainability profile appears in Figure 1.

## 3. Nutritional Characteristics of Insect Meals

### 3.1. Protein and Amino Acid Composition

Crude protein (CP) content varies considerably across insect species and shifts further with rearing substrate, larval stage, processing temperature, and defatting method. Full-fat BSF larval meal typically runs 35–45% CP on a dry matter basis, climbing to 55–65% once defatted [11,19]. *Tenebrio molitor* meal ranges from 45–60% CP in full-fat form, and *M. domestica* larval meal has been reported at 45–55% [3]. For comparison, high-quality soybean meal runs 44–48% CP and fishmeal 60–72%, which puts defatted insect meals in reasonably competitive territory. It bears emphasis, however, that crude protein concentration is a poor standalone indicator of nutritional value in monogastric nutrition; standardized ileal amino acid digestibility (SIAD) values, which account for the efficiency with which dietary amino acids are actually absorbed and made available for productive functions, provide a far more reliable basis for diet formulation [11,20,21]. SIAD data for insect meals are discussed in detail in Section 4.

**Figure 2:**
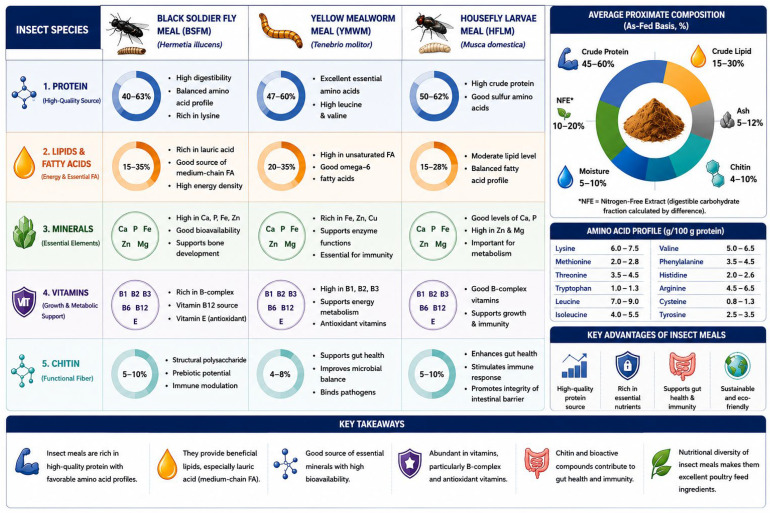
Nutritional Composition of Insect Meals: comparative overview of major nutritional components in commonly used insect meals for poultry nutrition. *NFE = Nitrogen-Free Extract, representing the digestible carbohydrate fraction of insect meal, calculated by difference after accounting for crude protein, crude lipid, crude fiber, ash, and moisture.

The amino acid profile is arguably insect meal’s strongest selling point. BSF larval meal supplies all the indispensable amino acids broilers need in roughly adequate proportion relative to lysine, though methionine tends to be first-limiting, a shortfall it shares with most plant proteins [20]. Lysine concentrations run 2.5–3.5% of DM, threonine 1.5–2.5%, and arginine 2.5–4.0%, figures that hold up well against fishmeal and comfortably beat cereal grains (Table 1). A comprehensive comparative overview of amino acid profiles and digestibility values across major insect species is provided in a recent synthesis by Naeem [9]. *T. molitor* meal stands out for its sulfur-containing amino acids: combined methionine and cysteine can reach 1.8–2.4% of DM [14], which makes *T. molitor* meal a plausible protein source in post-starter diets where methionine becomes limiting. Figure 2 summarizes the protein, lipid, mineral, vitamin, and chitin composition side by side.

### 3.2. Lipid Composition and Fatty Acid Profile

The lipid fraction of insect meal is both a nutritional asset and a potential liability, and which one it turns out to be depends heavily on whether the meal is full-fat or defatted. BSF larvae carry exceptionally high concentrations of medium-chain saturated fatty acids, most notably lauric acid (C12:0, 40–55% of total fatty acids under some substrate conditions) and myristic acid (C14:0, 7–15%) [11,22]. These medium-chain fats show antimicrobial activity against Gram-positive bacteria and certain lipid-enveloped viruses in laboratory settings, and they bypass hepatic portal circulation on absorption, getting oxidized quickly as an energy source [23]. Lauric acid’s antimicrobial action against *Clostridium perfringens*, *Salmonella* spp., and Campylobacter has been documented in in vitro systems [23]. It should be noted, however, that antimicrobial effects demonstrated in vitro may not translate directly to commercial broiler production conditions. Dietary lauric acid undergoes extensive digestion, emulsification, and absorption in the small intestine; the effective concentration at the intestinal mucosal surface under physiological conditions may differ substantially from in vitro exposure concentrations. Additional modifying factors (dietary fat composition, feed processing method, and competitive absorption by other lipids) further complicate extrapolation from in vitro to in vivo outcomes. In vivo studies specifically designed to confirm lauric acid-mediated antimicrobial effects at the intestinal level under commercial dietary conditions are therefore needed before this mechanism can be considered established in broilers.

*T. molitor* and *M. domestica*, by contrast, lean toward unsaturated fatty acids, linoleic acid (C18:2*n* − 6), oleic acid (C18:1*n* − 9), and variable amounts of α-linolenic acid (C18:3*n* − 3) depending on substrate [14]. That composition carries consequences for oxidative stability, both of the raw ingredient and of the meat it eventually becomes: higher polyunsaturated fatty acid (PUFA) content makes lipids more prone to peroxidation during storage and processing, which means diets built around large amounts of non-defatted insect meal need careful attention to antioxidant supplementation.

### 3.3. Chitin: Biology, Concentration, and Nutritional Implications

Chitin, a linear homopolymer of N-acetylglucosamine (GlcNAc) linked by β (1,4) glycosidic bonds, forms the structural backbone of insect cuticle, gut lining, and tracheal tubes. It is arguably the single most distinctive functional component of insect meal, absent from conventional feed protein sources and engaged in a web of interactions with both the avian gut and immune system. Chitin content ranges from around 2% in *M. domestica* to as much as 8–12% in whole BSF larval preparations; defatting and other processing steps that disrupt cuticle structure can lower extractable chitin, but they do not remove it entirely [24,25].

The nutritional story here is genuinely two-sided, and a balanced assessment must give equal weight to both dimensions. On the negative side, chitin functions as an indigestible structural polysaccharide that forms a physical barrier encasing cytoplasmic proteins and lipid droplets within the larval body, thereby limiting access by digestive proteases and lipases. This antinutritional effect is most pronounced in young chicks: gastric chitinase (acidic mammalian chitinase) activity in birds runs substantially below mammalian levels and is particularly low in the first two weeks post-hatch, when digestive enzyme secretion is still immature [24,26]. Studies consistently demonstrate that SIAD values for whole-larval preparations with intact chitin are 10–20 percentage points lower than for defatted, processed meals in which the chitin matrix has been disrupted, with the deficit most severe in starter-phase chicks (0–14 days post-hatch). At the same time, and on the positive side, chitin fragments, particularly short-chain, low-molecular-weight oligomers generated by partial enzymatic hydrolysis, act as prebiotics: they selectively encourage bifidobacteria and lactobacilli growth in the caecal microbiota, suppress putrefactive bacteria, and serve as ligands for pattern recognition receptors in the gut immune system [25]. Whether chitin nets out as a nutritional cost or an immunological benefit depends critically on inclusion level, bird age, and the degree of chitin depolymerization achieved by dietary chitinase activity; no single metric captures that balance adequately, and formulation decisions should account for both dimensions.

### 3.4. Mineral and Vitamin Content

Insect meals generally offer favorable mineral profiles. Calcium in full-fat BSF larval meal can run exceptionally high, up to 8–9% of DM when larvae are reared on calcium-rich substrates such as dairy-containing food waste, largely because calcium accumulates in the exoskeleton. That level comfortably exceeds broiler calcium requirements (roughly 0.9–1.0% of DM in starter diets) and can throw off dietary balance unless supplemental calcium carbonate is adjusted downward [11]. Phosphorus sits at a moderate 0.7–1.2% of DM, and the resulting calcium-to-phosphorus ratio in BSF meal can widen enough at high inclusion rates to risk metabolic bone disease if the Ca:P ratio is not actively managed.

Trace minerals, such as iron, zinc, and manganese, are generally adequate for broiler needs and comparable to soybean meal. The absence of phytic acid, a potent mineral-chelating compound found in plant proteins, is a genuine nutritional plus, though how much it actually improves mineral bioavailability relative to SBM-based diets has not been well quantified [3]. Vitamin B12 (cobalamin) and riboflavin show up in biologically meaningful amounts in most insect meal preparations, a trait that sets insect meal apart from plant protein and gives it some potential value in partially replacing both SBM and vitamin premix components.

**Table 1:** Comparative nutritional composition of major insect meals and conventional protein sources.

Parameter	BSF (Full-Fat)	BSF (Defatted)	*T. molitor*	*M. domestica*	Soybean Meal	Fishmeal
Crude protein (%DM)	38–45 ^a^	55–65 ^a^	48–62 ^b^	50–58 ^c^	44–48 ^d^	60–72 ^d^
Crude fat (%DM)	25–40 ^a^	7–15 ^a^	22–35 ^b^	15–28 ^c^	1–3 ^d^	8–12 ^d^
Lysine (%DM)	2.5–3.5 ^a^	3.5–4.5 ^a^	3.2–4.2 ^b^	3.0–4.0 ^c^	2.8–3.0 ^d^	4.5–5.5 ^d^
Met + Cys (%DM)	1.0–1.6 ^a^	1.5–2.0 ^a^	1.8–2.4 ^b^	1.5–2.2 ^b^	1.4–1.6 ^d^	2.0–2.8 ^d^
Lauric acid (%FA)	35–55 ^a^	30–50 ^a^	2–5 ^b^	3–8 ^b^	<1 ^d^	<1 ^d^
Chitin (%DM)	5–8 ^a^	4–7 ^a^	3–6 ^b^	1–3 ^c^	0	0
Calcium (%DM)	5–9 ^a^	4–8 ^a^	0.1–0.3 ^b^	0.3–0.8 ^b^	0.3 ^d^	2–5 ^d^
AMEn (MJ/kg DM)	13–18 ^a^	10–14 ^a^	14–18 ^b^	12–16 ^b^	10–12 ^d^	12–14 ^d^

BSF = black soldier fly (*H. illucens*); DM = dry matter; FA = fatty acids; AMEn = apparent metabolizable energy corrected for N retention. Values represent ranges from multiple peer-reviewed sources. Superscript letters indicate primary data sources: ^a^ = Schiavone et al. [11] and Marono et al. [19]; ^b^ = van Broekhoven et al. [14]; ^c^ = Makkar et al. [3]; ^d^ = standard NRC/industry reference values. Note: individual study values may fall outside the stated ranges depending on rearing substrate, larval stage, and processing method.

## 4. Digestibility and Nutrient Utilization

### 4.1. Protein and Amino Acid Digestibility

Standardized ileal amino acid digestibility (SIAD) is the appropriate benchmark for judging protein quality in monogastric animals, and it matters here precisely because crude protein content alone does not predict how well broilers will actually use that protein. Published SIAD values for BSF larval meal span an unusually wide range, from as low as 55% for some amino acids in whole-larval preparations with intact chitin, up to 88–92% in defatted, heat-processed meals, a spread that reflects just how sensitive digestibility is to processing [11,19,22].

Chitin appears to be the main culprit behind reduced protein digestibility in whole insect meal. It forms a structural scaffold that physically encases cytoplasmic proteins and fat droplets, limiting access for digestive enzymes. Break that matrix down, through mechanical processing, high-temperature drying, solvent defatting, or steam pelleting, and protein digestibility improves substantially, in some cases bringing defatted BSF meal’s SIAD values within a few percentage points of fishmeal [11]. Whole-dried larvae with minimal processing, in contrast, show markedly lower digestibility, particularly in younger birds where gastric chitinase activity is still limited [26].

Digestibility also varies meaningfully by amino acid within the same meal, which matters for diet formulation. Lysine SIAD in BSF larval meal ranges from 76% to 90% across published studies (roughly 82% on average), methionine SIAD runs somewhat higher (84–92%), and arginine SIAD often clears 90% in well-processed preparations [11,20]. Tryptophan has been characterized less thoroughly but seems to lag behind other essential amino acids in some preparations, plausibly because heat-induced Maillard reactions between reducing sugars and lysine’s epsilon-amino group interfere, a concern most relevant to high-temperature drying. Table 2 summarizes SIAD ranges for major amino acids in BSF larval meal and *T. molitor* meal alongside conventional protein source comparators.

### 4.2. Energy Utilization and Apparent Metabolizable Energy

Apparent metabolizable energy corrected for nitrogen retention (AMEn), the standard energy metric in poultry nutrition, tracks closely with fat content and digestibility in insect meal. Full-fat BSF larval meal typically delivers 13–18 MJ/kg DM, competitive with or better than full-fat soybean meal (roughly 10–12 MJ/kg DM), while defatted preparations drop to 10–14 MJ/kg DM depending on residual fat [11]. *T. molitor* meal, with its higher share of unsaturated fatty acids, tends to show somewhat higher AMEn relative to its caloric content, since unsaturated fats digest more efficiently than saturated ones in avian physiology.

Several studies have documented digestive enzyme adaptation to chitin-containing diets, upregulated gastric chitinase, increased proventricular lysozyme secretion, and stimulated intestinal brush-border enzyme activity among them [25,29]. That suggests the apparent antinutritional cost of chitin may ease somewhat as birds adjust to insect-based diets over time. Whether that adaptation happens fast enough to matter in commercial production, where flocks are typically raised for only 35–42 days, leaving little time for the gut to fully adjust, remains an open and practically important question.

### 4.3. Factors Modifying Digestibility

Beyond species and chitin content, several other factors shape digestibility outcomes. Inclusion rate behaves nonlinearly: several studies report comparable or better digestibility at moderate levels (5–10%) but a decline above 15%, possibly reflecting a dilution of digestive enzyme capacity or a chitin-load threshold effect on mucosal function [27]. Bird age is a consistent modifier too, with starter-phase chicks (0–14 days) showing lower digestibility than grower-phase birds, in line with their still-maturing enzyme secretion and shorter gut transit time. Defatting method matters as well; hexane extraction, supercritical CO_2_ extraction, and mechanical pressing each affect protein structure and residual fat differently, producing digestibility outcomes divergent enough to complicate meta-analysis. All of this points to a field badly in need of standardized protocols.

**Figure 3:**
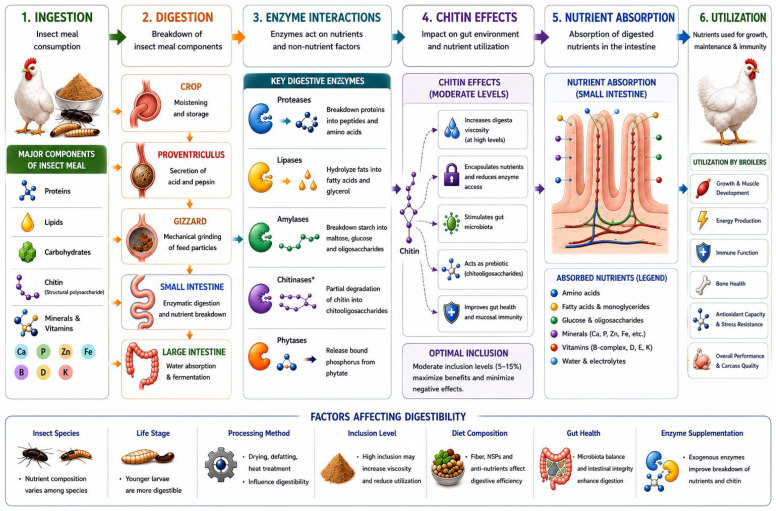
Digestibility Pathways of Insect Meals in Broilers. From ingestion to nutrient absorption: the roles of enzymes, chitin, and gut health.

Feed processing methods also exert a significant but frequently overlooked influence on digestibility. Extrusion processing, subjecting ingredients to combined high temperature, pressure, and mechanical shear, can effectively disrupt the chitin matrix and denature structural proteins, improving nutrient release in processed insect meal preparations. Pelleting temperature exerts a similar dual influence: high-temperature conditioning above 80 °C during pelleting may trigger Maillard reactions between reducing sugars and amino groups, reducing lysine bioavailability particularly. Reducing particle size through fine milling increases surface area contact with digestive enzymes and consistently improves nutrient bioavailability. Enzyme supplementation is an additional practical intervention; exogenous chitinase, protease, and lipase added to insect meal-containing diets has shown promise in several studies as a means of compensating for structural barriers chitin imposes, with chitinase supplementation in particular restoring digestibility closer to that of conventional protein sources. These processing and formulation variables collectively underscore the urgent need for standardized experimental protocols for reporting insect meal digestibility data, which the field currently lacks. Figure 3 outlines the digestibility pathway of insect meal in broilers, from ingestion through enzyme interaction, chitin effects, and nutrient absorption, to host utilization, along with the key factors that modify it.

## 5. Effects on Growth Performance and Carcass Traits

### 5.1. Body Weight Gain and Feed Intake

Whether insect-based protein can match conventional ingredients for growth performance is the central question driving applied research in this field, and it has now been tackled by enough controlled feeding trials to give a reasonably clear, if not entirely uniform, answer. Across studies replacing SBM or FM partially with BSF larval meal, the balance of evidence suggests inclusion up to 10–15% of diet DM supports growth broadly on par with control diets, with some trials reporting numerically better body weight gain (BWG) at 5–10% inclusion [11,28,30,31,32]. A recent meta-analysis of broiler carcass parameters confirmed that BSF meal inclusion at or below 10% was associated with maintained or improved performance, while inclusion above 15% produced significant reductions in carcass weight [33]. Quantitatively, mean BWG in broilers receiving 5–10% BSF larval meal typically falls within 97–105% of control diet values at day 35, with the majority of trials reporting no statistically significant difference from the SBM control at these inclusion levels [11,30,31,32]. Feed conversion ratio at similar inclusion levels is generally equivalent to or marginally improved relative to controls, with reported FCR values ranging from approximately 1.55 to 1.85 kg/kg depending on genotype, diet phase, and experimental conditions. At inclusion levels above 15%, BWG reductions of 5–12% relative to controls have been reported in several trials, though this threshold varies with amino acid supplementation, processing method, and bird age at first exposure [21].

Several mechanisms plausibly explain the growth benefits seen at moderate BSF inclusion, beyond simple protein swapping. Lauric acid’s antimicrobial activity against intestinal pathogens may lower subclinical enteric disease burden and free up nutrient absorption. Antimicrobial peptides, defensins and cecropins isolated from insect hemolymph and cuticle among them, suppress pathogenic Gram-positive and Gram-negative bacteria in vitro and ex vivo, hinting at a possible role as a partial stand-in for antibiotic growth promoters in antibiotic-free systems [34]. Chitin’s prebiotic stimulation of caecal microbiota may also boost short-chain fatty acid (SCFA) production, adding an energy substrate and supporting enterocyte health. Section 6 and Section 7 dig into these mechanisms in more depth.

The evidence for *T. molitor* meal is thinner but points in the same direction as BSF findings. Benzertiha et al. [31] found that replacing 5–10% of SBM with *T. molitor* meal held BWG and feed conversion ratio (FCR) steady relative to controls, with some improvement in nitrogen retention at the lower inclusion level. Push past 15%, though, and several mealworm trials report numerically worse BWG, which the authors attributed partly to elevated leucine interfering with isoleucine transport and partly to unidentified antinutritional factors tied to the mealworm exoskeleton. Housefly larval meal trials in East Asia show more scatter, some report better performance than SBM controls, and others show no difference, likely reflecting real variation in substrate, drying method, and inclusion level across studies run under different biosecurity conditions.

### 5.2. Feed Conversion Ratio

Feed conversion ratio, a composite measure of nutrient intake against deposition efficiency, follows a pattern broadly parallel to BWG across the insect meal literature. At moderate inclusion (5–15%), FCR in BSF-supplemented diets typically sits within 2–5% of control values, a gap that rarely reaches statistical significance given the sample sizes most published trials use. Several studies report numerically better FCR at 5–10% BSF inclusion without statistical confirmation, a pattern that keeps coming up and has drawn methodological criticism: typical poultry trials may simply be underpowered to detect biological effects that matter in practice [35].

### 5.3. Carcass Traits and Organ Development

Carcass yield generally holds steady across insect meal inclusion levels in studies that report it, provided BWG itself is not compromised. Dressing percentages of 72–76% have been reported in BSF-supplemented trials, indistinguishable from soybean meal controls in most cases [11,30]. Breast meat yield, the most commercially valuable cut, is sensitive to amino acid supply; below roughly 15% inclusion of full-fat BSF meal without supplementation, breast yield holds up fine; push past that without correcting amino acid supply, and small but statistically real reductions in breast percentage start showing up [28]. The recent trial by Tóth et al. [32] reported a significant increase in breast yield in birds fed full-fat *H. illucens* and *T. molitor* meals relative to the control, suggesting that well-formulated insect meal diets can improve breast muscle deposition at appropriate inclusion levels. Liver and spleen weight, expressed relative to body weight, offer a rough window into immune activation. Several studies report modest relative spleen enlargement in birds on insect meal diets, more likely a sign of immune stimulation via chitin and bioactive peptides than actual tissue damage [30,34].

## 6. Immunomodulatory Effects of Insect Meal

### 6.1. Innate Immunity: Pattern Recognition and Chitin-Mediated Signaling

Insect meal’s immunomodulatory properties are among the most scientifically distinctive, and practically consequential, aspects of its biology, and they deserve close examination. The chicken’s innate immune system, its first line of defense against pathogens and environmental challenge, works through pattern recognition receptors (PRRs) that detect conserved molecular signatures known as pathogen-associated and damage-associated molecular patterns (PAMPs and DAMPs). Toll-like receptors (TLRs), NOD-like receptors (NLRs), and C-type lectin receptors (CLRs) are the PRR families most relevant to insect meal, since several of its bioactive components, chitin, AMPs, and bioactive lipids, engage these receptor systems directly.

Chitin is recognized by several PRRs, including TLR2, Dectin-1, and the receptor for advanced glycation end-products (RAGE). Chitin particles behave differently depending on size. Low-molecular-weight fragments (roughly 3–8 GlcNAc units), generated by enzymatic hydrolysis in the gut, act as proinflammatory triggers, activating macrophages and dendritic cells through TLR2-dependent NF-κB signaling and driving production of IL-12, TNF-α, and reactive oxygen species [36,37]. High-molecular-weight chitin tends toward immunosuppressive or regulatory effects, stimulating anti-inflammatory cytokines like IL-10 and TGF-β through alternative macrophage activation (M2 polarization). It is important to note explicitly that much of the mechanistic evidence for TLR2, Dectin-1, and RAGE engagement by chitin derives from murine and human experimental systems [36,37]. Evidence directly characterizing these signaling pathways in avian-specific studies is more limited. TLR2 has been identified and functionally characterized in chickens, with demonstrated responses to similar PAMPs as mammalian TLR2; however, the full downstream signaling cascades and their relative immunological contributions in poultry have not been as thoroughly mapped as in mammalian models. The mechanistic conclusions presented here regarding chitin signaling pathways in broilers should therefore be understood as plausible extrapolations from mammalian research, with direct avian experimental validation remaining an important research priority.

### 6.2. Macrophage Polarization and Phagocytic Capacity

Macrophage activation is a central mechanism through which insect-derived bioactives shape the innate immune landscape [36,37]. Avian heterophils, functionally analogous to mammalian neutrophils, and monocyte-derived macrophages, which handle phagocytosis and antigen presentation, both shift phenotype depending on cytokine milieu and PRR stimulation. The classically activated (M1) phenotype, marked by strong phagocytic capacity, elevated respiratory burst, and proinflammatory cytokine output, gets a push from chitin-NF-κB signaling, LPS-TLR4 co-stimulation during concurrent bacterial challenge, and possibly the AMPs present in insect cuticle extracts.

Several in vitro studies using avian macrophage cell lines (HD11, MQ-NCSU) or primary monocyte-derived macrophages have shown that chitin particle preparations from insect cuticle raise phagocytic index, respiratory burst activity (measured by luminol-enhanced chemiluminescence), and nitric oxide production via iNOS upregulation [34,38]. It is essential to acknowledge, however, that the majority of macrophage activation evidence in this literature derives from in vitro studies using established avian cell lines or primary cells stimulated with purified chitin preparations under controlled conditions. In vivo confirmation of macrophage polarization status, phagocytic index, and respiratory burst activity in commercial broilers fed insect meal under field-representative conditions remains limited to a small number of studies, and the translation from in vitro activation data to in vivo immune competence cannot be assumed without direct experimental validation. The data currently available represent biologically plausible mechanistic evidence; they do not yet constitute proof of in vivo efficacy under commercial conditions.

### 6.3. Antimicrobial Peptides: Insect-Derived and Host-Induced

Insects carry their own sophisticated innate immune system that has co-evolved with microbial pathogens for hundreds of millions of years, generating a pharmacologically diverse set of AMPs with broad-spectrum activity. Defensins, cecropins, attacins, diptericins, proline-rich peptides, and lysozyme-like enzymes have all been isolated from *H. illucens*, *M. domestica*, *T. molitor*, and other species, collectively active against Gram-positive and Gram-negative bacteria, fungi, and some viruses [34,39,40]. Whether these peptides survive the thermal processing, drying, and pelleting that commercial feed production demands, with enough structural integrity left to matter biologically in the broiler gut, is a genuinely open and important question. Several groups have detected biologically active concentrations of defensin-like peptides and lysozyme in water-extracted fractions of commercially produced BSF larval meal, suggesting some thermal stability [39]. High-temperature drying above 80 °C or autoclaving substantially reduces AMP activity in most preparations tested [40].

Emerging processing technologies may partially resolve this bioactivity–biosafety tension. Microencapsulation of insect-derived AMP-rich fractions in lipid, protein, or polysaccharide matrices has shown promise in vitro for protecting peptide structural integrity during thermal processing and gastrointestinal transit. Spray-drying at controlled temperatures, combined with encapsulants such as maltodextrin or whey protein isolate, can substantially reduce thermal denaturation of labile bioactive peptides while maintaining their biological activity upon dissolution at the target site. While these approaches have not yet been validated specifically for insect AMPs in commercial feed manufacturing settings, they represent a technically feasible direction that warrants direct investigation, particularly given the trade-off between biosafety requirements (which favor high-temperature treatment) and functional bioactivity preservation (which favors milder processing).

Beyond the AMPs insects bring with them, insect meal consumption may also prompt the broiler’s own intestinal epithelial cells to ramp up production of host AMPs. Cathelicidins and gallinacins, the principal AMP families in chickens, are encoded by 14 avian β-defensin (AvBD) genes. Several studies report AvBD gene expression rising in the jejunal and ileal mucosa of broilers fed BSF or *T. molitor* meal compared with SBM controls, an effect that may run through chitin-TLR2 signaling in gut epithelial cells or MyD88-dependent pathways [38].

### 6.4. Adaptive Immunity: Antibody Production and Lymphocyte Responses

Adaptive immunity, marked by antigen-specific lymphocyte responses, immunological memory, and class-switched antibody production, has drawn less attention in insect meal research than innate immunity, largely because adaptive immune assays are more complex and costly, and broiler trials tend to run short. Even so, a body of evidence has accumulated showing that insect meal feeding shifts both humoral and cell-mediated adaptive immunity in measurable ways.

Several trials have measured immunoglobulin concentrations, IgY (the avian analog of mammalian IgG), IgA, and IgM, in serum and intestinal secretions. Benzertiha et al. [31] reported significantly higher serum IgY in broilers fed BSF larval meal at 5% inclusion compared with SBM controls at 35 days, an effect that disappeared at higher inclusion levels, suggesting a hormetic or threshold-dependent response. Secretory IgA (sIgA) in intestinal lavage fluid, functionally important for mucosal immunity, was elevated in birds fed moderate chitin-containing insect meal across several studies, consistent with chitin-driven activation of the gut immune network [27,38]. Flow cytometry-based analysis suggests BSF meal at moderate levels (5–10%) is associated with numerically higher proportions of CD4+ helper and CD8+ cytotoxic T cells in splenic lymphocyte populations relative to SBM controls, though most studies lack statistical power to confirm the trend [34].

Beyond immunoglobulin concentration measurements, adaptive immune evaluation should ideally extend to vaccine responsiveness and pathogen challenge models, which offer functional readouts more directly relevant to field conditions. The hemagglutination inhibition (HI) titer response to Newcastle disease virus vaccination, ELISA-based response to infectious bursal disease virus antigen, and antibody titres following Salmonella challenge all represent validated functional endpoints more clinically meaningful than isolated Ig concentration measurements. de Souza Vilela et al. [34] demonstrated that BSF-supplemented diets modulated both innate and adaptive immune responses in a disease-relevant broiler context. Further well-designed challenge model studies incorporating vaccine response endpoints would substantially strengthen the evidence base for insect meal’s immunological benefits and bridge the gap between biomarker data and clinically meaningful resistance outcomes.

### 6.5. Cytokine Regulation and Inflammatory Balance

The cytokine profile in broiler intestinal tissue and systemic circulation reflects how innate and adaptive immune signaling integrate, and it offers a mechanistically useful readout of immune competence and inflammatory tone. Several insect meal studies have measured intestinal or splenic cytokine gene expression by real-time qPCR, targeting IL-1β, IL-6, IL-10, IL-12, IFN-γ, and TNF-α. Despite considerable variation in study design, a consistent pattern emerges: moderate insect meal inclusion nudges the intestinal cytokine profile toward a regulated proinflammatory state, modest upregulation of IL-1β and IL-12 (favoring Th1 responses) alongside maintained or elevated IL-10, an anti-inflammatory regulator, a combination consistent with balanced immunocompetence rather than pathological inflammation [31,38].

### 6.6. Lauric Acid and Bioactive Lipids as Immunomodulatory Agents

The fatty acid profile of BSF larval meal matters immunologically well beyond its antimicrobial role. Medium-chain fatty acids, lauric acid in particular, act on macrophage function through several routes at once: they blunt NF-κB activation downstream of LPS-TLR4 stimulation, cutting proinflammatory cytokine output during Gram-negative bacterial challenge; they promote PPARγ activation in macrophages and enterocytes, switching on an anti-inflammatory transcriptional program; and they appear to suppress COX-2 expression in stimulated macrophages, lowering prostaglandin E_2_ output [23]. That combination, direct antimicrobial action against luminal pathogens paired with host immunomodulatory effects that keep inflammation in check, makes lauric acid a genuinely versatile bioactive with real potential in antibiotic-free broiler production. The effect sizes for key immunological indicators in broiler chickens fed insect meal diets were summarized in Table 3.

## 7. Gut Health, Microbiome, and Host–Microbe Interactions

### 7.1. Gut Morphology and Intestinal Integrity

The gastrointestinal tract of the broiler chicken is not merely a passive conduit for nutrient absorption but a dynamic immunophysiological interface at which dietary inputs, luminal microorganisms, and mucosal immune cells engage in continuous and mutually regulatory crosstalk. The morphological architecture of the small intestinal mucosa, measured by villus height (VH), crypt depth (CD), and the VH:CD ratio, provides an integrated assessment of mucosal health that reflects the balance between absorptive capacity (principally determined by VH), epithelial renewal rate (reflected in CD), and the net mucosal surface area available for nutrient uptake. Several insect meal studies have measured intestinal morphometric parameters, with consistently interesting findings.

Biasato et al. [27,30] conducted systematic morphometric analysis of the duodenum, jejunum, and ileum of broilers consuming BSF larval meal at inclusion levels ranging from 5% to 15%, reporting significantly greater jejunal VH and VH:CD ratios in the 5–10% groups compared with SBM-based controls at day 35. These findings have been largely corroborated by Dabbou et al. [29], who reported improved VH in the duodenum and jejunum of BSF-supplemented birds, and by several other groups examining *T. molitor* and housefly larval meal. The mechanisms responsible for villus elongation associated with moderate insect meal consumption likely include: (1) prebiotic stimulation of lactobacilli and bifidobacteria that produce SCFA, particularly butyrate, which promotes enterocyte proliferation and inhibits apoptosis; (2) reduced colonization by pathogenic bacteria, including *Clostridium perfringens* and Campylobacter, that damage villus epithelium; (3) lauric acid-mediated antimicrobial effects that reduce luminal pathogen pressure; and (4) AMP-stimulated upregulation of epithelial renewal processes.

### 7.2. Tight Junction Proteins and Paracellular Permeability

Intestinal barrier integrity is determined in large part by tight junction proteins (TJPs), including claudin-1, claudin-3, claudin-5, occludin, zonula occludens-1 (ZO-1), and junctional adhesion molecules (JAMs). Moderate BSF larval meal inclusion (5–10%) is associated with upregulation of claudin-1, occludin, and ZO-1 mRNA expression in the jejunal mucosa relative to controls, suggesting enhanced epithelial barrier function [27,38]. The mechanistic basis is plausibly linked to butyrate-mediated stimulation of TJP gene expression and to the direct membrane-stabilizing effects of medium-chain fatty acids on lipid bilayer organization.

An important caveat applies to the majority of this TJP literature: improvements have predominantly been demonstrated at the mRNA level by qPCR. Whether these transcriptional changes translate to measurable increases in TJP protein abundance, confirmed by Western blotting or immunofluorescence, or, more importantly, to functional improvements in intestinal barrier permeability as assessed by transepithelial electrical resistance in ex vivo Ussing chamber models, or by in vivo lactulose:mannitol ratio measurements, has been confirmed in only a minority of published studies. Until protein-level and functional permeability data are systematically collected alongside gene expression data in the same experimental animals, the interpretation of mRNA-based TJP findings should remain appropriately cautious.

### 7.3. Mucin Production and Goblet Cell Dynamics

The mucus layer overlying the intestinal epithelium serves as a critical physical and biochemical barrier that excludes luminal pathogens from the epithelial surface, provides a habitat for mucosal commensal bacteria, and participates in innate immune signaling through the lectin-binding properties of mucin glycoproteins. Goblet cells, the principal secretory epithelial cell type producing mucins, are maintained at a density and activation state that reflects the interaction between dietary components, microbial stimuli, and mucosal immune signals. Several groups have documented increased goblet cell density in the jejunal and ileal mucosa of broilers consuming insect meal diets, particularly in the 5–10% inclusion range, a finding consistent with stimulated mucin production [27,29].

The mucin subtypes most relevant to intestinal protection are the gel-forming mucins MUC2 (principally expressed in the small intestine) and MUC5AC and MUC5B (more prominent in the trachea and stomach). MUC2 gene expression in the jejunum has been reported as significantly upregulated in BSF-supplemented broilers in at least two independent studies, suggesting not merely a numerical increase in goblet cells but enhanced secretory activity per cell [27]. Whether this represents an adaptive response to chitin-increased luminal viscosity or a genuinely immunoprotective mucin elaboration is an interpretive question that current data cannot fully resolve.

### 7.4. Intestinal Microbiome Modulation

The intestinal microbiota of the broiler chicken, a complex, dynamic community dominated by Firmicutes, Bacteroidetes, and Proteobacteria with distinct compositional profiles along the gastrointestinal axis, is profoundly influenced by dietary composition, and insect meal is no exception. A growing body of 16S rRNA amplicon sequencing and metagenomic data from insect meal trials has revealed consistent compositional shifts, though the specific genera and phyla affected vary across studies in ways that reflect differences in basal diet, insect species, inclusion level, and host genetics.

Among the most consistently reported effects of BSF larval meal inclusion on the caecal microbiome is a reduction in the relative abundance of *Clostridium perfringens* and related pathogenic Clostridiaceae, consistent with the antimicrobial effects of lauric acid and AMPs against Gram-positive bacteria, and an increase in beneficial genera including *Lactobacillus*, *Bifidobacterium*, and *Faecalibacterium prausnitzii*, a major butyrate producer and anti-inflammatory commensal [30,38]. These shifts are consistent with what would be anticipated from a chitin prebiotic effect, as chitin oligomers have been shown in in vitro fermentation models to selectively stimulate the growth of bifidobacteria and lactobacilli through a mechanism involving bacterial N-acetylglucosamine uptake systems [41]. Increased alpha-diversity metrics have been reported in several insect meal trials, suggesting a broader and potentially more functionally redundant microbial community associated with greater resilience to perturbation [30].

A significant gap in the current microbiome literature concerns functional characterization. Compositional data from 16S rRNA sequencing describe community membership but say little directly about what those bacteria are doing metabolically. Shotgun metagenomics, characterizing the full functional gene complement of the community, and metatranscriptomics, identifying actively transcribed microbial genes under specific dietary conditions, are beginning to be applied in nutritional intervention studies but remain largely absent from insect meal research specifically. Microbial metabolomics, profiling the full suite of small-molecule metabolites produced by the caecal community including SCFA, bile acids, tryptophan catabolites, and phenolic acids, would provide the most direct functional readout of microbiome activity and its relationship to host immunity and performance. Furthermore, the distinction between ileal and caecal microbiome responses to insect meal deserves more systematic attention, as these two compartments differ substantially in their microbial composition, dominant fermentation substrates, and immunological relationships with the host epithelium. Integrating microbiome functional profiling with host transcriptomics in the same experimental animals represents the most powerful path forward for establishing mechanistic causality [9,21].

### 7.5. Short-Chain Fatty Acids and the Gut–Immune Axis

SCFA, principally acetate, propionate, and butyrate, produced through microbial fermentation of dietary fibre and prebiotic substrates in the caecum and large intestine represent the primary currency through which the intestinal microbiota exerts systemic metabolic and immune effects. Butyrate, produced primarily by *Faecalibacterium prausnitzii*, *Roseburia intestinalis*, and members of the Lachnospiraceae family, is of particular importance in the context of poultry gut health because it serves as the principal energy substrate for colonocytes, promotes goblet cell mucin secretion, upregulates TJP expression via histone deacetylase (HDAC) inhibition, and inhibits NF-κB activation in intestinal epithelial cells, collectively conferring barrier-protective and anti-inflammatory effects [42].

**Figure 4:**
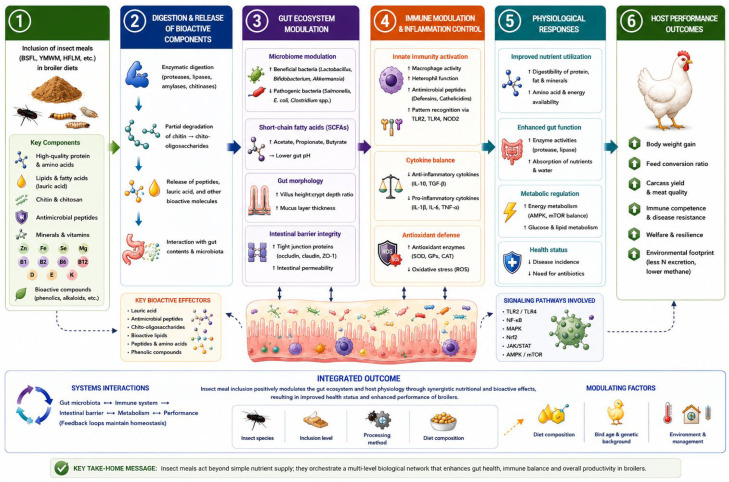
Mechanistic Framework Linking Insect Meal Inclusion to Gut Health and Host Performance in Broilers. ↑ means Increase; ↓ means decrease.

Several insect meal studies have measured caecal SCFA concentrations directly by GC-MS or HPLC, reporting higher total SCFA concentrations in birds receiving moderate BSF or *T. molitor* meal compared with SBM-based controls, with the butyrate fraction showing the largest absolute increases [27,38]. These findings are consistent with, and suggestive of, a mechanistic link between the prebiotic activity of chitin oligomers, stimulating butyrogenic bacteria, and improved barrier integrity and reduced inflammatory signaling. However, the relationship between increased caecal SCFA concentrations and improved productive performance remains largely associative in the current literature. Direct experimental evidence establishing causation, such as studies using SCFA synthesis-blocking interventions, microbiota transplantation models, or germ-free broiler systems, is currently lacking. The gut–immune axis narrative described here represents a biologically compelling working hypothesis supported by converging indirect evidence; it warrants rigorous causal testing before being treated as an established mechanistic pathway. Figure 4 illustrates the mechanistic framework linking insect meal inclusion to gut health and host performance in broilers.

## 8. Meat Quality, Product Safety, and Consumer Acceptance

### 8.1. Meat Quality Traits

Consumer acceptance of poultry products derived from insect-fed broilers is contingent not only on the nutritional performance of the birds but on the organoleptic and physicochemical properties of the meat itself. The primary parameters evaluated include breast meat color (CIE L*, a*, b*), pH, water-holding capacity (WHC), drip loss, shear force, and fatty acid profile. Results across the published literature are broadly reassuring for the commercial viability of insect-fed broiler products. Breast meat color values have come back statistically indistinguishable from SBM controls in most studies examining BSF or *T. molitor* meal at up to 15% of diet DM [11,30,32]. Ultimate pH stays consistently within the normal range (5.7–6.0) in insect meal trials, with no evidence of PSE or DFD meat anomalies linked to insect meal *per se*.

Consumer sensory evaluation of meat from insect-fed broilers has been addressed in a growing number of studies. Trained and consumer sensory panels rating appearance, flavor, texture, and overall acceptability have generally not been able to distinguish breast meat from BSF-supplemented broilers from conventional controls at blind evaluation, provided inclusion levels fall within the 5–15% range [35]. The modest increase in saturated medium-chain fatty acids associated with BSF-supplemented diets does not appear to translate into detectable flavor differences under standard cooking conditions. Consumer willingness-to-pay (WTP) studies in European markets have shown that explicit labeling of products as “from insect-fed chickens” can reduce purchase intention in some consumer segments, while framing the same product as “sustainably produced” tends to partially offset that reduction [35]. These findings highlight the critical importance of communication strategy in the commercial rollout of insect-fed poultry products.

### 8.2. Fatty Acid Composition of Meat

Because poultry metabolism deposits dietary fatty acids into muscle lipid without much endogenous desaturation, insect meal’s fatty acid profile measurably shapes muscle tissue fatty acid composition. Several studies report higher lauric and myristic acid concentrations in the breast and thigh meat of BSF-supplemented broilers relative to SBM controls, with the effect scaling with inclusion level [11,30]. A recent meta-analysis confirmed that these compositional shifts influence meat physicochemical parameters in a dose-dependent manner [33]. Higher lauric and myristic acid levels improve lipid oxidative stability (measured by TBARS/malondialdehyde equivalents), extend shelf life in refrigerated and frozen storage, and stabilize flavor during cooking—all commercially useful traits.

### 8.3. Safety Concerns and Regulatory Landscape

Safety evaluation of insect meal as a poultry feed ingredient covers several distinct concerns: microbiological contamination, heavy metal accumulation, pesticide and pharmaceutical residues, allergenicity, and prion risk. EFSA’s risk assessments of BSF larval meal [15,43] concluded that, produced under Good Manufacturing Practice on defined, safe substrates, insect meals pose no greater microbiological or chemical risk than conventional protein sources. The 2021 EU regulation (EU 2017/893, as amended) restricts what substrates can be used for commercial insect rearing, ruling out ruminant manure and catering waste among other high-risk materials while permitting vegetable matter, former foodstuffs, and certain processed crop residues, conditions under which safety has been well characterized.

Allergenicity is worth a specific mention, since BSF larval protein shares structural and epitope similarities with crustacean tropomyosin, raising a possible cross-reactivity risk for people with shellfish allergies. That concern is relevant when insects are eaten directly as human food, but EFSA considers it low for meat from insect-fed poultry, given no real evidence that insect-derived allergenic proteins transfer meaningfully into poultry muscle. Even so, consumer advocacy groups increasingly push for transparent labeling of insect-fed animal products, and evolving EU and other regulatory frameworks are starting to reflect that pressure.

### 8.4. Consumer Perceptions and Market Acceptance

Consumer attitudes toward insect-fed animal products matter for commercial viability in ways that production data alone cannot capture. Sogari et al. [35] and subsequent consumer research describe a consistent pattern: initial neophobia toward insect-derived products, driven largely by disgust responses that are cultural and psychological rather than rational. Indirect exposure, eating poultry or aquaculture products from insect-fed animals without knowing the feed source, generally produces neutral-to-positive sensory ratings indistinguishable from conventional controls. That distinction matters for how insect meal gets marketed commercially: the real acceptance barrier sits at the level of labeling and communication, not any intrinsic difference in product quality.

## 9. Economic and Industrial Feasibility

### 9.1. Production Costs and Economic Competitiveness

Insect meal’s commercial viability as a feed ingredient is currently held back by production costs well above soybean meal’s and only competitive with the higher end of the fishmeal market. BSF larval meal production cost estimates range roughly from $0.8 to $2.5 per kilogram of dried meal, depending on location, rearing scale, substrate cost, and energy input, against SBM spot prices typically running $0.4–$0.7 per kilogram and standard fishmeal at $1.5–$2.5 per kilogram [17,44]. At today’s production costs, insect meal competes economically with fishmeal but not with SBM in most global markets, which is why practical adoption so far has centered on replacing FM in broiler starter diets, where amino acid quality and growth factor benefits justify the premium.

Labor (substrate prep, larval harvest, processing), energy (climate control, drying), and capital amortization are the main cost drivers in insect production. Several techno-economic analyses project that industrial-scale BSF operations, above 10,000 tonnes of dried meal annually, could reach cost parity with fishmeal within 5–10 years through economies of scale, process automation, and co-product revenue (insect fat for feed or biodiesel, frass fertilizer, chitin extract) [44,45]. European companies, Protix (Netherlands), Agroprotect, Entomo Farms, and Ynsect (France) among them, have raised substantial venture capital betting on exactly this trajectory, though commercial-scale unit economics remain hard to verify without published financial data from operating facilities.

### 9.2. Regulatory Frameworks and Market Access

Regulation of insect meal in animal feed varies considerably by region. The European Union has moved fastest, issuing a series of regulations that progressively widened permissible species and applications: Regulation (EU) 2017/893 first permitted BSF, housefly, mealworm, morio worm, field cricket, tropical house cricket, and migratory locust processed animal proteins in aquaculture feed, and the 2021 amendment extended that to poultry and pig feed, opening a substantially larger market. North American regulators, the FDA and CFIA, have moved more slowly, requiring comprehensive safety dossiers under Generally Recognized as Safe (GRAS) determinations or new animal food ingredient petitions. Asian frameworks vary widely: China, Japan, and South Korea have preliminary rules in place, while many smaller markets have no specific provisions at all.

### 9.3. Scalability and Commercialization Challenges

Beyond cost and regulation, scaling up insect production raises real engineering and biological challenges still unresolved at commercial scale. Keeping substrate supply consistent, especially for operations relying on food waste or processing residues, demands sophisticated logistics and quality control. Disease management in dense larval rearing raises biosecurity issues not unlike those in intensive livestock farming. Substrate-to-meal conversion efficiency grows more variable at large scale than under controlled research conditions, which complicates cost projections built on small-scale data. Seasonal availability of certain waste substrates in some markets adds further variability to production throughput. None of these problems are unsolvable in principle, but all demand capital and operational expertise still in short supply across the agri-food sector.

## 10. Contradictions, Knowledge Gaps, and Research Limitations

### 10.1. Methodological Inconsistencies and Standardization Gaps

A clear-eyed look at the insect meal literature reveals methodological inconsistency substantial enough to limit how confidently conclusions can be drawn. The biggest sources of trouble: variation in insect species, larval stage, and rearing substrate across studies, which produces ingredient profiles that proximate analysis alone cannot capture; the absence of standardized processing parameters, drying temperature, defatting protocol, particle size, with most studies offering only general descriptions that make replication difficult; wide variation in experimental design around basal diet composition, replacement strategy (isonitrogenous, isocaloric, or simple percentage substitution), and control selection; small experiments, typically 6–8 replicate pens per treatment, that lack the statistical power to detect moderate but biologically meaningful differences; and short trial durations, frequently 35–42 days, that leave no room to characterize adaptation dynamics or chronic effects.

### 10.2. Conflicting Evidence on Growth Performance

The most practically consequential disagreement in the literature concerns growth performance at higher inclusion levels (above 15% of diet DM). Most studies report growth depression at these levels, but the proposed mechanisms do not agree: some authors point to amino acid limitation, methionine and isoleucine deficiency especially, at high BSF inclusion without supplementation; others blame chitin’s antinutritional effects on digestibility; still others invoke uncharacterized antinutritional factors or palatability issues that cut voluntary feed intake. Without dose-controlled, mechanistically designed studies that isolate these confounders systematically, there is no way to know which explanation dominates, and therefore which fix, amino acid supplementation, defatting, partial chitin removal, would do the most to raise the practical inclusion ceiling.

### 10.3. Knowledge Gaps in Immunological Mechanisms

The immunological evidence base is mechanistically promising but suffers from a near-total absence of in vivo studies that actually demonstrate improved pathogen resistance in insect meal-fed broilers under controlled challenge conditions. Most immune biomarker studies measure surrogate endpoints, cytokine gene expression, immunoglobulin levels, lymphocyte subset ratios, without showing that these intermediate changes translate into altered susceptibility to clinically important pathogens like *Salmonella enteritidis*, *Campylobacter jejuni*, *Clostridium perfringens*, or Eimeria species. Challenge model experiments under commercial-representative conditions are badly needed to move the field from mechanistic hypothesis to evidence-based recommendation. Nobody has yet parsed, in a properly factorial design, how much of the overall immunomodulatory effect comes from chitin versus AMPs versus lauric acid versus other bioactives, leaving the question of which component drives the effect still open.

### 10.4. Microbiome Research Limitations

Microbiome studies in insect meal trials have relied mostly on 16S rRNA amplicon sequencing targeting the V3–V4 region, which resolves taxonomy to genus level for common gut bacteria but says nothing about metabolic function or rare organisms. Shotgun metagenomics and metatranscriptomics, which would provide functional microbiome profiles, show up in only a handful of published insect meal studies, and metabolomic characterization of SCFA, bile acid, and aromatic amino acid metabolites has received even less attention. The result is that current microbiome data describe compositional shifts in isolation from the metabolic and immunological readouts that would establish whether those shifts actually matter functionally. Integrating microbiome composition with host transcriptomics and metabolite profiling in the same animals, a systems biology approach already used productively elsewhere in nutrition research, remains largely absent from insect meal studies.

## 11. Future Research Directions

### 11.1. Omics Integration and Mechanistic Resolution

The most pressing priority for the next decade of insect meal research is applying multi-omics frameworks—genomics, transcriptomics, proteomics, metabolomics, and microbiomics—to mechanistically resolve how insect-derived bioactives interact with the broiler host. Nutrigenomic approaches, characterizing genome-wide transcriptional responses in intestinal epithelium, immune tissue, and liver after insect meal feeding, could identify the molecular pathways these bioactives switch on or off. Integrating host transcriptomics with caecal metatranscriptomics and metabolomics in the same animals would allow multi-layer analysis of the host–microbiome–diet network, a level of integration insect meal research has not yet reached [21].

### 11.2. Precision Insect Farming and Bioactive Compound Optimization

Precision insect farming, tailoring nutritional and bioactive composition through controlled rearing conditions, substrate engineering, and environmental optimization, is a frontier that has received limited attention so far but holds real transformative potential. BSF larvae’s substrate plasticity opens the door to manipulating fatty acid profile, AMP content, chitin concentration, and amino acid composition by adjusting rearing substrate, temperature, humidity, photoperiod, and harvest timing. Preliminary controlled-substrate trials suggest that supplementing BSF rearing substrate with omega-3-rich materials, flaxseed or microalgae, substantially raises *n* − 3 PUFA in larval biomass, potentially enabling insect meal with an improved *n* − 3:*n* − 6 ratio for health-oriented broiler products [11]. Substrate supplementation with specific antimicrobial or vitamin precursors might similarly boost the functional bioactivity of insect meal preparations beyond what current general-purpose substrate formulations achieve.

### 11.3. AI-Assisted Feed Formulation and Digital Nutrition Platforms

Artificial intelligence and machine learning hold genuine promise for insect meal integration into commercial feed formulation. Current linear programming-based formulation systems optimize least-cost diets from ingredient composition and price matrices, but cannot capture the nonlinear, interaction-dependent biological effects of bioactive compounds across ingredient combinations. Machine learning models trained on large, multi-site datasets spanning growth performance, gut health, immunological, and microbiome endpoints could, in principle, identify optimal inclusion levels and synergistic ingredient combinations with far more precision than conventional trial-and-error. Digital twin modeling of broiler gut function, integrating mechanistic models of nutrient digestion, microbial fermentation, and immune activation, could offer in silico platforms for predicting biological responses to new insect-based formulations before committing to costly animal trials, speeding the path from research to commercial application considerably.

### 11.4. Climate-Resilient and Antibiotic-Free Production Systems

Where insect meal’s immunomodulatory properties intersect with the global push toward antibiotic stewardship and climate resilience deserves dedicated research attention. Insect meal’s capacity to modulate innate and adaptive immunity, support beneficial microbiota, and supply endogenous antimicrobial compounds makes it a potentially valuable tool for antibiotic-free broiler production, a market segment growing quickly under regulatory pressure and consumer demand across Europe, North America, and increasingly Asia. That said, the evidence for insect meal as an effective substitute for antibiotic growth promoters under realistic commercial challenge conditions, where birds face multiple pathogens, immunosuppressive viruses like infectious bursal disease virus and Marek’s disease virus, and environmental stress simultaneously, is not yet strong enough to support firm recommendations. Well-designed challenge studies in commercial-representative settings, benchmarked head-to-head against established alternatives (organic acids, phytogenics, zinc-based products, direct-fed microbials), are needed to establish insect meal’s real value in antibiotic-free programs.

### 11.5. Standardization, Regulatory Harmonization, and Life-Cycle Assessment

Faster scientific and commercial progress with insect meal depends critically on standardized protocols for ingredient characterization, feeding trial design, and reporting. Reference composition values, along the lines of NRC nutrient requirement tables for poultry, for the major insect meal categories (full-fat BSF, defatted BSF, *T. molitor*, *M. domestica*) would meaningfully improve how comparable and interpretable future studies are. International collaboration among research institutions, industry, and regulators on standardizing LCA methodology is equally essential for robust comparisons of insect meal’s environmental sustainability across production contexts and regions. The Global Platform for Sustainable Natural Ingredients and the International Platform of Insects for Food and Feed (IPIFF) are emerging bodies that could help drive this harmonization, though their progress so far has been limited by resource constraints and the divergent interests of member organizations.

## 12. Conclusions

The evidence reviewed here supports a carefully qualified, but substantively optimistic, assessment of insect meal as a sustainable protein ingredient in broiler nutrition. The nutritional composition of the major commercial species, defatted BSF larval meal and *T. molitor* meal especially, compares favorably with conventional protein sources in crude protein and amino acid profile, with functional bioactives that plant and marine alternatives simply lack. Growth performance at moderate inclusion (5–15%) generally matches SBM- or FM-based controls in well-designed trials, and several mechanistically plausible pathways, antimicrobial fatty acid effects, AMP-mediated pathogen suppression, chitin prebiotic stimulation, and enhanced barrier integrity, link insect meal consumption to measurable gains in gut health and immune competence.

The immunomodulatory evidence, while mechanistically coherent and biologically interesting, still needs considerably stronger in vivo validation under commercial challenge conditions before it can support firm practical recommendations. The gut microbiome data are compositionally compelling but metabolically underexplored. The sustainability case for insect meal is strong against fishmeal; against SBM, it depends heavily on production context. The economic case rests on continued scaling of production infrastructure and cost trajectories that look plausible but remain unproven at commercial scale.

In practical terms, insect meal is most defensibly used as a partial replacement (5–10%) for fishmeal or high-quality animal protein in broiler starter diets, where its amino acid profile and bioactive properties best match host needs and production challenges. Inclusion in SBM-based diets works at similar levels with attention to amino acid supplementation, calcium-phosphorus balance, and processing quality. Full replacement of either SBM or FM as the sole protein source is not supported by current evidence at any life stage without substantial reformulation and supplementation.

The research priorities most likely to move this field forward are: standardized production and characterization protocols; mechanistically designed challenge studies evaluating pathogen resistance; multi-omics integration in feeding trials to unpack host–microbiome–diet interactions; precision insect farming to optimize bioactive compound profiles; and rigorous life-cycle analysis built on standardized boundaries and transparent assumptions. Regulatory harmonization across major producing and consuming regions, enabling free movement of safely produced insect meal and insect-fed animal products, is a precondition for the market scale that would, in turn, drive the cost reductions mainstream adoption needs.

Insect meal is neither a universal fix for poultry protein’s sustainability problems nor a marginal academic curiosity. It is a scientifically promising, commercially developing, and ecologically coherent ingredient that has earned a legitimate and growing place among the sustainable protein sources available to the broiler industry. How quickly its potential gets realized will depend less on the underlying biology, which already has a credible foundation, than on regulatory alignment, investment, cost reduction, and consumer communication, the pieces that let a circular bioeconomy actually operate at the scale global poultry production demands.

## Figures and Tables

**Table 2:** Comparative standardized ileal amino acid digestibility (SIAD, %) values for major insect meals and conventional protein sources in broiler chickens.

Amino Acid	BSF Larval Meal (Full-Fat)	BSF Larval Meal (Defatted)	*T. molitor* Meal	Soybean Meal	Fishmeal
Lysine	76–83	82–90	80–88	89–93	88–93
Methionine	84–89	87–92	85–91	88–93	88–92
Threonine	74–82	80–87	78–86	82–88	84–90
Tryptophan	68–75	74–80	72–82	85–91	83–89
Isoleucine	78–85	83–88	80–87	88–93	87–92
Valine	77–84	81–87	80–87	86–92	87–91
Leucine	79–86	83–89	81–88	87–92	87–92
Arginine	82–89	88–93	84–91	90–95	90–94
Cysteine	67–78	72–82	71–80	83–89	80–87

BSF = black soldier fly (*H. illucens*); *T. molitor* = yellow mealworm. Values represent approximate ranges compiled from published literature [11,20,21,27,28]. Ranges reflect variation due to processing method, defatting, larval stage, and bird age. Defatted BSF preparations consistently show higher SIAD than full-fat preparations, particularly for lysine and methionine. Authors are advised to verify specific values against the cited primary sources before using them in diet formulation matrices.

**Table 3:** Summary of reported effect sizes for key immunological indicators in broiler chickens fed insect meal diets (predominantly BSF larval meal, 5–10% inclusion level).

Immune Indicator	Insect Species/Inclusion	Direction	Approximate Effect Size	Representative Reference(s)
Serum IgY	BSF, 5%	↑ significant	+15–28% vs. control	[31]
Serum IgY	BSF, >10–15%	No change or ↓	NS or −5 to −10%	[31]
Intestinal sIgA	BSF/YMW, 5–10%	↑	+12–35% vs. control	[27,38]
IL-1β gene expression	BSF, 5–10%	↑ modest (Th1 priming)	+1.3–2.1-fold	[31,38]
IL-10 gene expression	BSF/YMW, 5–10%	↑ (anti-inflammatory)	+1.4–2.5-fold	[31,38]
IL-12 gene expression	BSF, 5%	↑	+1.5–2.2-fold	[38]
TNF-α	BSF, 5–10%	Variable	↑ in some studies; NS in others	[34,38]
Phagocytic index (macrophage)	BSF, 5–7.5%	↑	+15–40% vs. control	[34,38]
Respiratory burst (NO)	BSF, 5–10%	↑	+20–45% vs. control	[38]
CD4+ T cells (splenic)	BSF, 5–10%	↑ numerically	+5–12% (mostly NS)	[34]
PHA proliferation (PBMCs)	BSF, 7.5%	↑ significant	Significant at 7.5%; NS at 15%	[27]
Spleen weight (relative)	BSF, 5–15%	↑ modest	Modest enlargement; not pathological	[30,34]

BSF = black soldier fly (*H. illucens*); YMW = yellow mealworm (*T. molitor*); IgY = immunoglobulin Y; sIgA = secretory immunoglobulin A; IL = interleukin; TNF = tumor necrosis factor; PHA = phytohemagglutinin; PBMCs = peripheral blood mononuclear cells; NS = not statistically significant. Effect sizes are approximate ranges compiled from studies cited in the text; values may vary depending on insect species, processing method, bird genotype, and experimental conditions. Authors are advised to consult primary sources before citing specific numerical values. ↑ means increase, while ↓ means decrease.

## Data Availability

No new data were generated or analyzed in this study. All information discussed in this review is derived from previously published articles, which are cited throughout the manuscript.
